# The Impact of Cross-Linking Effect on the Space Charge Characteristics of Cross-Linked Polyethylene with Different Degrees of Cross-Linking under Strong Direct Current Electric Field

**DOI:** 10.3390/polym11071149

**Published:** 2019-07-04

**Authors:** Shuchao Wang, Quan Zhou, Ruijin Liao, Lai Xing, Nengcheng Wu, Qian Jiang

**Affiliations:** 1 State Key Laboratory of Transmission & Distribution Equipment and Power System Safety and New Technology (Ministry of Education), School of Electrical Engineering, Chongqing University, Chongqing 400044, China; 2 Deyang Electric Power Supply Company of State Grid Corporation, Deyang 618000, China

**Keywords:** cross-linked polyethylene (XLPE), low-density polyethylene (LDPE), space charge, cross-linking degree, pulsed electro-acoustic method (PEA)

## Abstract

Cross-linked polyethylene (XLPE) obtained by the crossing-linking reaction of polyethylene (PE) can greatly enhance the mechanical properties and other properties of PE, which makes XLPE widely applied in the field of electric power engineering. However, the space charges can distort the distribution of the electrical field strength in the XLPE applied in the insulation materials, which can shorten the service life of the insulation materials. Therefore, the space charge characteristics of XLPE under the strong direct current (DC) electric field have been the focus of scholars and engineers all over the world. This article has studied the impact of the cross-linking effect on the space charge characteristics of XLPE with different degrees of cross-linking. For this issue, we used dicumyl peroxide (DCP) as the cross-linking agent and low-density polyethylene (LDPE) as the base material for the preparation of samples. Besides, the space charge distribution was measured by the pulsed electro-acoustic method (PEA). In addition, the average charge density as a characteristic parameter was introduced into the experiment, which was used to quantitatively analyze the impact of the cross-linking effect on the space charge characteristics of XLPE with different degrees of cross-linking. Meanwhile, we also explained the impact of the cross-linking effect on XLPE with different degrees of cross-linking from a microscopic point of view. Ultimately, some important conclusions can be obtained. For instance, the cross-linking effect significantly increases the threshold electrical field strength of XLPE, and as the content of cross-linking agent increases, the threshold electrical field strength increases at first and then decreases, and the threshold electrical field strength reaches the maximum value when the content of the cross-linking agent is 1.0% or 2.1%. Besides, the cross-linking effect introduces negative charge traps into the LDPE and increases the densities of the deeper charge traps, and so on. In addition, we have also analyzed the average charge density, and we have summarized the theoretical model of the average charge decay, namely, $Q(t)=Q_{0}+\alpha e^{-\frac{t}{\beta }}$, which is very effective for explaining the dissipation characteristics (more conclusive contents can be seen in the conclusion section of this article).

## 1. Introduction

Cross-linked polyethylene (XLPE) is obtained by the cross-linking reaction of polyethylene (PE). XLPE, compared to PE, shows better mechanical properties and other properties. Therefore, in the global power industry, XLPE has quickly occupied an important position since its emergence and become the main material for the insulation of power equipment and cables. However, XLPE still has various problems, which need to be solved by scholars and engineers. One of the more prominent problems is that the space charges can distort the distribution of electric field strength in the insulation materials that are used for power equipment and cables. Therefore, this can shorten the service life of the power insulation materials [1,2,3,4]. Therefore, the space charge characteristics of XLPE under the strong direct current (DC) electric field have always been the focus of scholars and engineers at home and abroad.

In recent years, Meunier et al. in the United Kingdom [5] used molecular simulation methods for the study of electron traps in PE. They found that PE’s electron affinity increases as the bending degree of the PE chain increases, and the PE chains in the crystal zone are mainly linear chains arranged in a regular manner, while the PE chains in the amorphous zone are curved chains. In addition, Choo et al. [6] studied the space charge accumulation characteristics of the XLPE cable body in the temperature gradient field under the DC electric field. The research has shown that the charges are more easily injected into XLPE and more easily transported inside the XLPE as the temperature increases, and at the same temperature, the negative charges move faster than the positive charges. Also, when the cable temperature decreases from the inside to the outside, the maximum electrical field strength increases near the outer shield of the cable with the increase of the temperature gradient field. The experimental team composed by Fu et al. [7] studied the formation of the space charges in the body of an XLPE cable in the voltage reversal and the temperature gradient field; the space charges cause the electric field to change. The research has shown that the “mirror effect” of the space charges during the voltage reversal is at a stable state, which is not affected by the type of the charges and the dynamic process of the charge formation. Vissouvanadin et al. [8] in France studied the effect of polarizable impurities on the space charge electric field distribution in high-voltage DC cable polymer insulation materials. This study has shown that the space charges generated by the polarization of cross-linking by-product impurities cause a severe distortion of the electric field in XLPE, resulting in a significant increase in the local electric field. At the same time, Teyssedre et al. [9] summarized the research status of polymer dielectrics and predicted the future development direction. They believed that combining microdescription with macroscopic means, the establishment of the charge transport models in dielectric bodies, and the aging caused by the space charges can be the focus of future research. In Japan, Sekii et al. [10] studied the generation and dissipation of the negatively-polar space charges in XLPE and ethylene propylene rubber (EPR). This study has shown that the negatively polar space charges in XLPE are caused by cross-linking by-products, and the heteropolar charges in XLPE that are undried and free of antioxidants come from the combination of acetophenone and water. In addition, Japanese scholar Toshikatsu [11] systematically studied the electrical properties of various aspects of XLPE/SiO_2_ nanocomposite composite media. Fabiani studied the space charge packets in the XLPE DC electric field [12] in Italy. The study showed that there are two kinds of space charge packets in XLPE, which are fast and slow space charge packets. Mauanti [13] of Italy proposed a charge transport model based on the pulsed electro-acoustic method (PEA) method and the space charge characteristics of XLPE, and estimated the apparent mobility and charge trap levels of the charges according to the model. The research team composed by Rogti et al. [14] studied the dynamic characteristics of space charges on the interface of XLPE under the DC electric field at different temperatures. The study has found that the positive charge injection occurred in all the tests, and the injected charges were affected by the temperature and the electric field strength. At the same time, the positive charges were stored on two identical XLPE interfaces for a longer period of time. In addition, they also studied the physical interface of the electrode/XLPE and XLPE/XLPE under a DC electric field. The research has shown that both aluminum and gold electrodes can be injected with electrons and holes, but the injection of aluminum electrodes is much easier. Besides, the semiconducting electrodes of the mixture of carbon black and PE are easier to be injected into the electrons [15].

Many scholars at home and abroad have done a lot of research work on the modification of PE insulation materials, such as heat treatment to eliminate cross-linking by-products, the introduction of additives to prevent the accumulation of space charges, and so on. However, most of the research focuses on the description of macroscopic phenomena, and there is the lack of in-depth discussion and analysis of the microscopic causes of such macroscopic phenomena. At present, few scholars have conducted in-depth analysis on the impact of the cross-linking effect on the space charge characteristics of XLPE with different cross-linking degrees. However, the cross-linking effect can greatly affect the performance of XLPE with different cross-linking degrees. The impact of the cross-linking effect on the space charge characteristics of XLPE with different cross-linking degrees is the major research content of this article, which includes the impact of the cross-linking effect on the charge injection and accumulation characteristics of XLPE with different cross-linking degrees and the impact of the cross-linking effect on the charge dissipation characteristics of XLPE with different cross-linking degrees. This article discusses the reasons for this macro effect from the micro level. Finally, the related conclusions from qualitative and quantitative aspects are obtained.

## 2. Materials and Methods 

### 2.1. Samples Preparation

#### 2.1.1. The Preparation Principle of XLPE

Peroxide cross-linking, also known as chemical cross-linking, is a series of free radical reactions initiated by the decomposition of peroxides at high temperature; thereby, this leads to the cross-linking reaction of PE. When the cross-linking agent is simple peroxide, the reaction process is as follows:

The peroxide is thermally decomposed to form free radicals:

ROOR → 2RO

Free radicals attack PE macromolecular chains and capture hydrogen atoms in the molecular chain, which ultimately forms PE macromolecular chain free radicals:

RO·+ —CH_2_—CH_2_—CH_2_— → ROH + —CH_2_—CH—CH_2_—

PE macromolecular chain free radicals have a high degree of reaction flexibility. When two PE molecular chain free radicals meet, they combine with each other to form a chemical bond of the polymer chain, thereby ultimately leading to cross-linking:

$$\begin{array}{rr} 2—{\mathrm{CH}}_{2}—\mathrm{CH}\;—{\mathrm{CH}}_{2}—\to & —{\mathrm{CH}}_{2}—\mathrm{CH}—{\mathrm{CH}}_{2}—\\ & |\;\;\;\;\;\;\\ & —{\mathrm{CH}}_{2}—\mathrm{CH}—{\mathrm{CH}}_{2}— \end{array}$$

When the peroxide-containing mixture is processed, the temperature cannot be too high. In order to prevent pre-cross-linking, the processing temperature must be lower than the decomposition temperature of the peroxide. When dicumyl peroxide (DCP) is used as the cross-linking agent, the safe processing temperature is 120 °C. The heat treatment must be carried out during cable insulation extrusion during the production process; however, the material temperature cannot be higher than the DCP decomposition temperature. Then, the heat treatment is performed to cross-link PE [3,14,15].

#### 2.1.2. The Preparation of Samples Used in This Experiment

##### Raw Materials

The experimental base selected for this experiment was low-density polyethylene (LDPE) particles with the density distribution of 0.910–0.925mg/mm^3^, the melt index of 2.1 to 2.2, and the melting point of 120 °C.

The cross-linking agent for this experiment was dicumyl peroxide, C_18_H_22_O_2_, which is called vulcanizing agent DCP and DCP*. The relative molecular mass of dicumyl peroxide is 270.37, and dicumyl peroxide is a white crystal with the melting point of 41–42 °C, the relative density of 1.082, and the decomposition temperature of 120–125 °C. Besides, dicumyl peroxide is very stable at room temperature, and when dicumyl peroxide is irradiated with light, dicumyl peroxide can gradually turn yellowish. In addition, dicumyl peroxide is insoluble in water, but it is soluble in ethanol, ether, acetic acid, benzene, and petroleum ether.

##### Samples Preparation Process

The preparation process of XLPE samples with different cross-linking degrees:

1. Manufacture of Particles

The LDPE and the corresponding mass of the cross-linking agent DCP were uniformly mixed in a molten state at a certain temperature to achieve the purpose of producing particles. This experimental temperature required both LDPE and DCP to be melted, but the cross-linking agent has not yet been decomposed, and the cross-linking reaction has not yet occurred. LDPE with 0%DCP (Pure LDPE) has the melting point of 112 °C, and the melting point of the cross-linking agent is 41 °C to 42 °C; at the same time, the decomposition temperature of the cross-linking agent is 120 °C to 125 °C. Therefore, the cross-linking agent DCP with different levels and pure LDPE were mixed uniformly at 116 °C to achieve the purpose of producing particles [7].

2. Cross-Linking

The materials obtained in the previous step were preheated for 10 min on a flat vulcanizing machine at 120 °C; during this time, the vulcanizing press was not pressurized. Then, at a temperature of 180 °C, the flat vulcanizing machine was pressurized to cause a cross-linking reaction of the materials. At this time, the pressure was maintained at 15MPa, and the cross-linking time was 20 min. Finally, the samples were taken out from the flat vulcanizing machine, and the samples were cooled to room temperature.

The specific process of samples preparation was as follows:

1. Weighing a certain amount of pure LDPE and the corresponding mass of DCP, and grinding the DCP into powders with the ball mill.
2. Mixing DCP and LDPE on an auto-measured twin-screw extruder at 116 °C to produce particles. Ultimately, the particle materials with DCP mass fractions of 0%, 1%, 2.1%, 3%, and 5% are prepared, respectively.
3. Preheating the particle materials for 10 min on the flat vulcanizing machine at 120 °C; during this time, the vulcanizing press is not pressurized. Then, the temperature is raised to 180 °C, and, at this time, the pressure is maintained at 15 MPa for 20 min to fully cross-link the samples.
4. Removing the samples from the flat vulcanizing machine and allowing samples to cool to achieve the room temperature, thereby obtaining XLPE samples with different cross-linking degrees. The sample sizes are 4 cm × 4 cm × 0.16 mm and 4 cm × 4 cm × 0.1mm.

##### Samples Pretreatment

The cross-linking agent used in the preparation of XLPE material was DCP. At high temperatures, DCP can decompose and make the LDPE cross-link to form XLPE. Cross-linking by-products such as cumyl alcohol and acetophenone produced by DCP decomposition existed as impurities in XLPE. These impurities were decomposed into ion pairs at the higher electric field. These ion pairs migrated to the two ends of the electrode under the action of the electric field, and eventually, it formed opposite polarity charges near the electrode/insulation interface. In order to eliminate the effect of cross-linking by-products, the samples can be pretreated by the degassing method; namely, the samples were placed in the vacuum oven, respectively, for heat treatment for a certain period of time to eliminate residual and volatile cross-linking by-products [6,7].

In this paper, we study the impact of the cross-linking effect on the space charge characteristics of LDPE, and at the same time eliminate the interference of thermal stress, water vapor, acetophenone, and other cross-linking by-products, which were produced by samples preparation on the space charge characteristics [7,16]. The prepared samples were pretreated by vacuum drying and degassing treatment. The samples were placed in the vacuum drying oven to degas and dry for 48h. The temperature of the vacuum drying oven was 80 °C, and the pressure of the vacuum drying oven was 50 Pa.

### 2.2. The Space Charge Testing System

**Figure 1:**
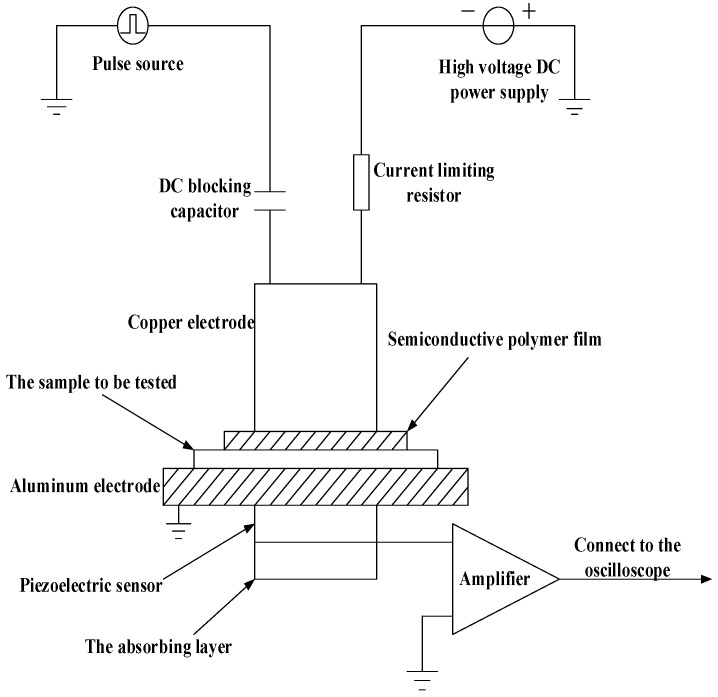
The space charge testing system.

In this experiment, the space charge distribution of the samples was measured by the PEA method. The space charge measurement system is shown in Figure 1 [17,18].

In this space charge testing system, the output voltage range of the high voltage DC source in this system is 0–20kV; at the same time, the pulse source output voltage range of this system is 0–0.2kV, and the pulse width of this system is 5ns. In addition, the thickness of piezoelectric sensor is 25μm. The upper electrode is a copper electrode, and a semiconductive polymer film is between the upper electrode and the sample. The lower electrode is an aluminum electrode, and the absorption layer is under the piezoelectric film, and the right side of the absorption layer is an amplifier; in addition, silicone oil is used as an acoustic coupling agent. Besides, the LeCory7200 digital oscilloscope, which establishes real-time communication with the computer for data storage and analysis, is used to collect measurement signals.

### 2.3. The Experimental Method

#### 2.3.1. The Threshold Field Strength of the Charge Injection

In this paper, the step-up voltage experiment was used to determine the field strength of each sample when the charges started to be injected, namely, the threshold field strength.

This basic principle [17,19] is that the charge density at the interface between the sample and the electrode—namely, the output voltage at the electrode position—should increase linearly with the increase of the applied voltage when there is no charge injection. In addition, the charge density value no longer increases linearly with the applied voltage, and a point deviates from the linear growth when the charge injection occurs, which can determine the space charge injection threshold field strength of the samples.

The specific operation process was to boost the voltage from 0 kV to 6 kV, and the interval of the boosted voltage was 0.2 kV. As the applied voltage increases, the output voltage value at the position of the corresponding electrode on the oscilloscope was recorded.

#### 2.3.2. The Impact of Cross-Linking Effect on Charge Injection and Accumulation Characteristics of XLPE

In order to obtain the impact of the cross-linking effect on the charge injection and accumulation characteristics of XLPE with different cross-linking degrees, we used the samples with the size of 4cm×4cm×0.16mm, and the voltage of 1kV was applied on the samples to measure the reference signals, respectively. Then, these samples were applied to the voltage for one hour at the electric field strength of 30 kV/mm, 50 kV/mm, and 80 kV/mm, respectively. PEA testing was carried out at 0 min, 1 min, 3 min, 5 min, 10 min, 20 min, 30 min, and 60 min during the process of applying the voltage. Therefore, the space charge distribution in each sample was obtained.

#### 2.3.3. The Impact of Cross-Linking Effect on the Charge Dissipation Characteristics of XLPE

In order to obtain the impact of the cross-linking effect on the charge injection and accumulation characteristics of XLPE with different cross-linking degrees, we used the samples with the size of 4cm×4cm×0.16mm, and the voltage of 1kV was applied on the samples to measure the reference signals, respectively. Then, these samples were applied to the voltage for one hour at the electric field strength of 30 kV/mm, 50 kV/mm, and 80 kV/mm, respectively. Then, the applied electric field was removed, and the upper and lower electrodes were shorted and grounded, and the time of removing the voltage was 20min. PEA testing was carried out at 0 min, 1 min, 2 min, 5 min, 10 min, and 20 min during the process of removing the voltage; thereby, the space charge distribution in each sample was obtained. 

#### 2.3.4. The Impact of Cross-Linking Effect on the Average Charge Density of XLPE

The previous experiments were mainly for qualitative analysis. However, we would like to further analyze the impact of the cross-linking effect on the space charge characteristics of XLPE. Then, it is necessary to further quantitatively analyze the measured space charge distribution data to extract the relevant characteristic parameters of the space charge distribution in the applied voltage experiment and the dissipation experiment. To this end, the average charge density q(t) was used for further analysis in this paper [13,20].

(1) $$q\left(t\right)=\frac{1}{L}\int_{0}^{L}|q_{p}\left(x,t\right)|dx$$

In this formula, $q_{p}\left(x,t\right)$ is the space charge density at the position x in the thickness direction of the sample at time *t*; L is the sample thickness; and q(t) is the average density of the space charge in the sample at time *t*.

## 3. Results and Discussion

### 3.1. The Threshold Field Strength of the Charge Injection

**Figure 2:**
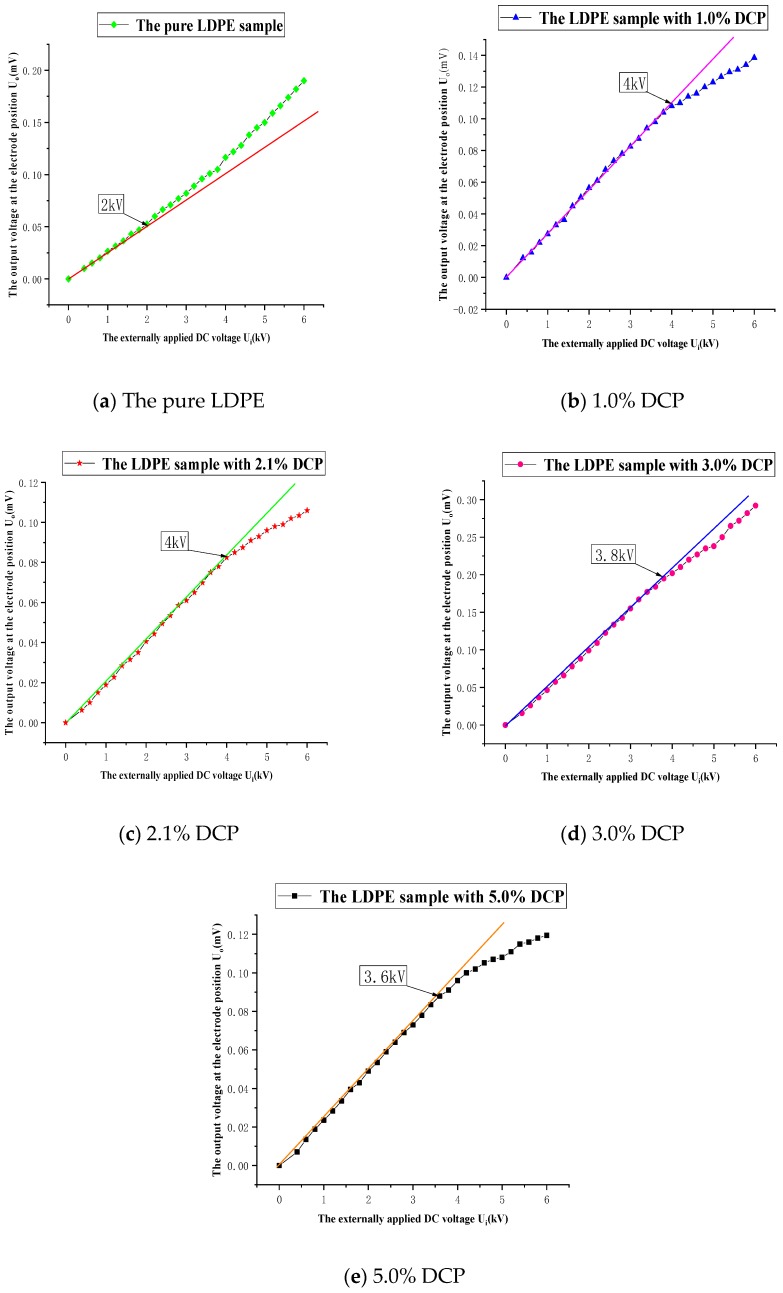
The experimental results of the stepwise voltage enhancement of the samples prepared by different contents of cross-linking agents. (**a**) The experimental result of the pure sample. (**b**) The experimental result of the sample with 1.0% dicumyl peroxide (DCP). (**c**) The experimental result of the sample with 2.1% DCP. (**d**) The experimental result of the sample with 3.0% DCP. (**e**) The experimental result of the sample with 5.0% DCP.

The experimental results of the stepwise voltage enhancement of the samples prepared by different contents of cross-linking agents are shown in Figure 2.

It can be seen from Figure 2 that as the applied voltage increases, the output voltage value at the corresponding electrode position on the oscilloscope tends to increase, and the output voltage value increases linearly at the initial stage when the applied voltage increases. However, when the applied voltage increases to a certain value, the output voltage no longer increases linearly, and the output voltage no longer increases linearly, which indicates that charge injection begins to occur at the interface position. The applied voltage value corresponding to this point is the voltage value at which the space charges start to be injected, and it can be seen from Figure 2 that the applied voltage value basically increases first and then decreases.

**Figure 3:**
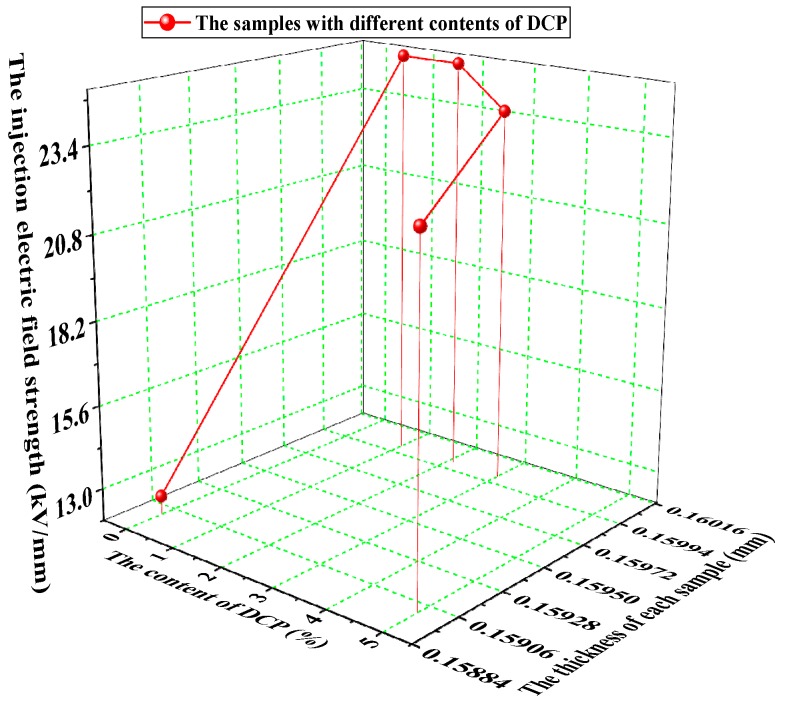
The threshold field strength of samples with different contents of DCP (Red dots represent the samples with different contents of DCP).

In order to eliminate the influence of the thickness difference of each sample to make the data more comparable, the values of the charge injection threshold electric field strength of these samples with different contents of cross-linking agents are shown in Figure 3. These values are obtained by dividing the voltage values at the start of the space charges by the actual thicknesses of the samples.

It can be seen from Figure 3 that the threshold electric field strength of each sample after cross-linking is greatly improved compared with the pure LDPE samples. The highest values of the electric field strength of the samples with 1.0% DCP and 2.1% DCP are both 25 kV/mm. It can also be seen from Figure 3 that the injection threshold electric field strength of the sample with 5% DCP is significantly reduced, which has a value of 22.64 kV/mm, but it is still much higher than that of the pure LDPE, which has a value of 12.58 kV/mm. These results indicate that the cross-linking effect significantly increases the threshold electric field strength of XLPE with different degrees of cross-linking. As the content of the cross-linking agent increases, the threshold electric field strength increases first and then decreases.

### 3.2. The Impact of the Cross-Linking Effect on the Charge Injection and Accumulation Characteristics of XLPE

#### 3.2.1. The Space Charge Injection and Accumulation Characteristics at the Electric Field Strength of 30kV/mm

**Figure 4:**
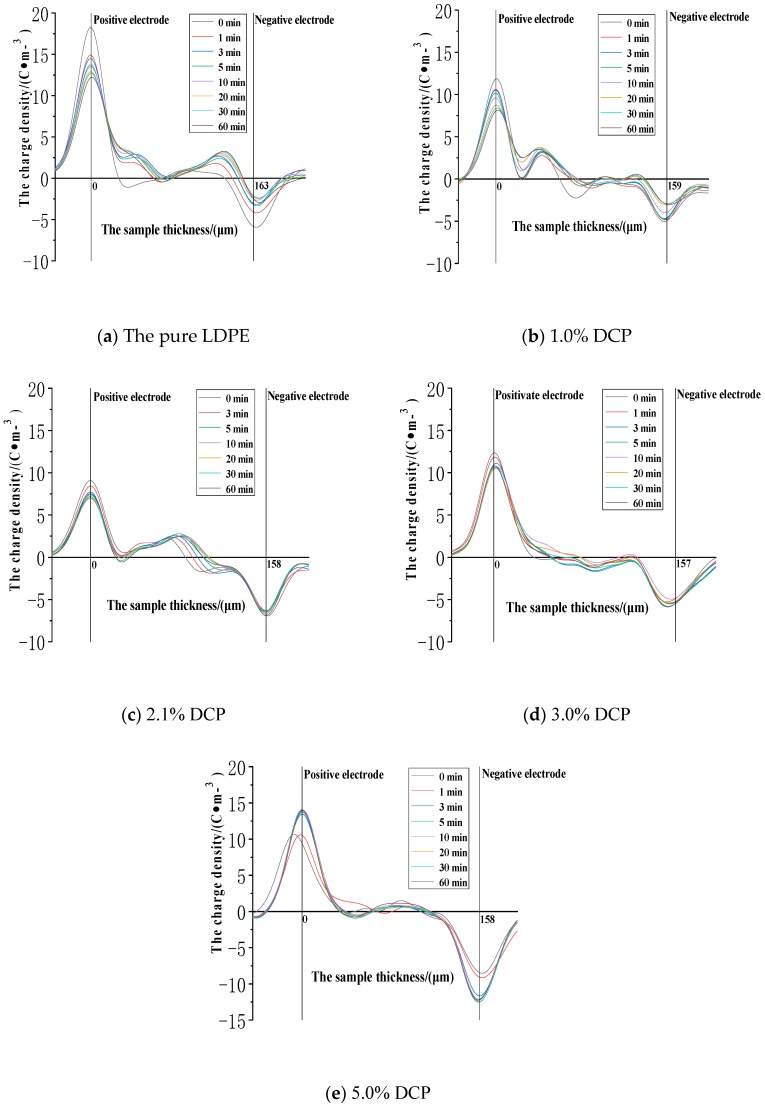
The space charge distribution when voltage is applied to each sample at the electric field strength of 30kV/mm. (**a**) The experimental results of the pure LDPE samples. (**b**) The experimental results of the samples with 1.0% DCP. (**c**) The experimental results of the samples with 2.1% DCP. (**d**) The experimental results of the samples with 3.0% DCP. (**e**) The experimental results of the samples with 5.0% DCP. LDPE: low-density polyethylene.

The space charge distribution at the electric field strength of 30 kV/mm in the applied voltage experiment is shown in Figure 4.

It can be seen from Figure 4 that the peak values of the charge densities at the positions of the two electrodes in each sample basically decrease as the applied voltage time increases. The significant charge injection can be seen in the sample near the two electrodes, and the charge density value is gradually increased. Although the charge value in the middle of the sample is also increased, the number of charges in the middle of the samples is still relatively small. The fluctuation in the early stage of the applied voltage is large, but it is basically stable after half an hour. After the voltage is applied, the charges are gradually injected and accumulated in the samples. In the pure LDPE, when the voltage is applied (0 min), a small number of heteropolar charges accumulate at the positions of the two electrodes. However, after the initial stage of voltage application, the polarities of the accumulated charges in the sample are all positive, and most of them accumulate in the vicinity of the two electrodes. At the same time, the charge densities in the middle of the samples are low. In the LDPE after cross-linking, the negative space charge peak appears in the sample during the entire applied voltage phase. Compared with the pure LDPE, the positive charge peak near the negative electrode is significantly reduced or even becomes the negative peak after cross-linking, and the negative charge peak appears for a long time near the positive electrode. This indicates that the cross-linking effect causes the injection and accumulation of the negative space charges. The charge density of a large part of the pure LDPE is relatively high, and the charge density in a large area of the middle part of the sample after cross-linking is at a relatively low level. This indicates that the cross-linking effect makes the charges less likely to be injected and migrated into the interior of the medium. At the electric field strength of 30kV/mm, the charge density at the anode position is at a level of 15 C∙m^−3^. The charge density value at the anode position generally decreases first and then increases with the increase of the content of the cross-linking agent. Among them, the value of the pure LDPE is 18.19C∙m^−3^, and the lowest is the value of the sample with 2.1% DCP, which is 9.16 C∙m^−3^, and the corresponding value of the sample with 5% DCP is 13.94 C∙m^−3^. The charge density values of the first four samples at the cathode position are all about −6 C∙m^−3^, while the charge density at the cathode position of this sample with 5% DCP reaches −12.61 C∙m^−3^. 

#### 3.2.2. The Space Charge Injection and Accumulation Characteristics at the Electric Field Strength of 50 kV/mm

**Figure 5:**
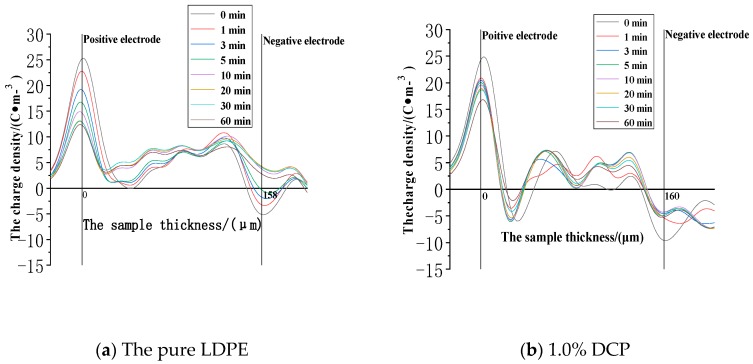

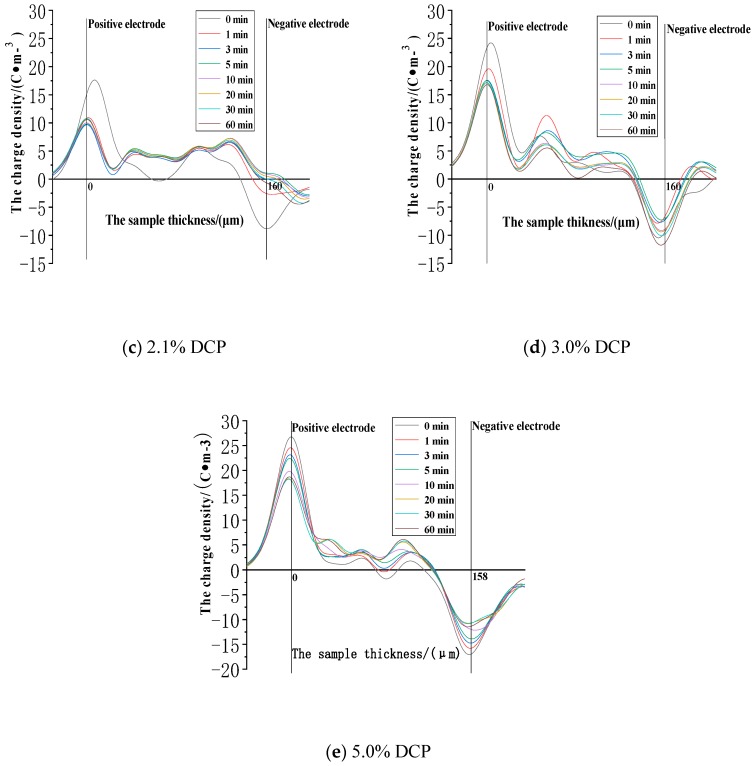
The space charge distribution when voltage is applied to each sample at the electric field strength of 50kV/mm. (**a**) The experimental results of the pure LDPE samples. (**b**) The experimental results of the samples with 1.0% DCP. (**c**) The experimental results of the samples with 2.1% DCP. (**d**) The experimental results of the samples with 3.0% DCP. (**e**) The experimental results of the samples with 5.0% DCP.

The space charge distribution at the electric field strength of 50 kV/mm in the applied voltage experiment is shown in Figure 5.

It can be seen from Figure 5 that the value of the charge density at the positive electrode position, compared with the charge density of 15 C∙m^−3^ at the position of the positive electrode at a field strength of 30 kV/mm, is greatly improved to about 25 C∙m^−3^, and the value of the charge density at the cathode position is also increased from 6 C∙m^−3^ to approximate 10 C∙m^−3^. As the voltage application time increases, it can be clearly seen that the charge density values at the two electrode positions are both decreased, while the charge density values inside the sample are gradually increased, which can be because the charges at the electrode positions are gradually injected into the medium through the interface between the electrode and the medium. After the charges are injected into the medium, they are first captured by the charge traps closer to the interface. At this time, the charges trapped by the traps can have a certain shielding effect on the electrodes, so that the intensities of the combined electric field at the electrode positions are lower, resulting in the gradual decrease in the peak value of the charge density at the electrode position. As the applied voltage experiment continues, the charges trapped by the charge traps near the interface can deviate from the traps due to the disturbances of some external and internal factors. At the same time, under the action of the electric field force, the charges can move deeper into the medium, causing the charge density value inside the sample to increase slowly, which indicates that the charge injection and accumulation do occur in the applied voltage experiment. In fact, the charge distribution at the electric field strength of 50 kV/mm is very different from the charge distribution at the electric field strength of 30 kV/mm. At 30kV/mm, the charges are mainly distributed near the electrodes, and compared with the positions of the electrodes, the charge accumulation is small, and the charges are low in a large region inside the medium. However, a large number of charges accumulate in a large region inside the medium at the electric field strength of 50 kV/mm. And the charge density value in this region and the charge density value at the electrode position reach a comparable level. This feature is most prominent in the pure LDPE.

#### 3.2.3. The Space Charge Injection and Accumulation Characteristics at the Electric Field Strength of 80 kV/mm

**Figure 6:**
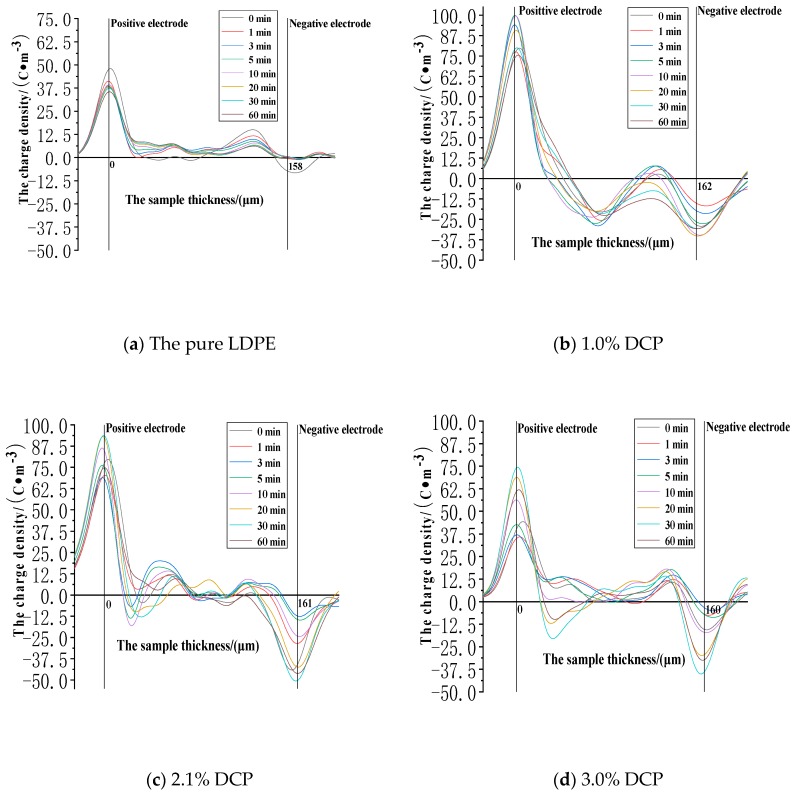

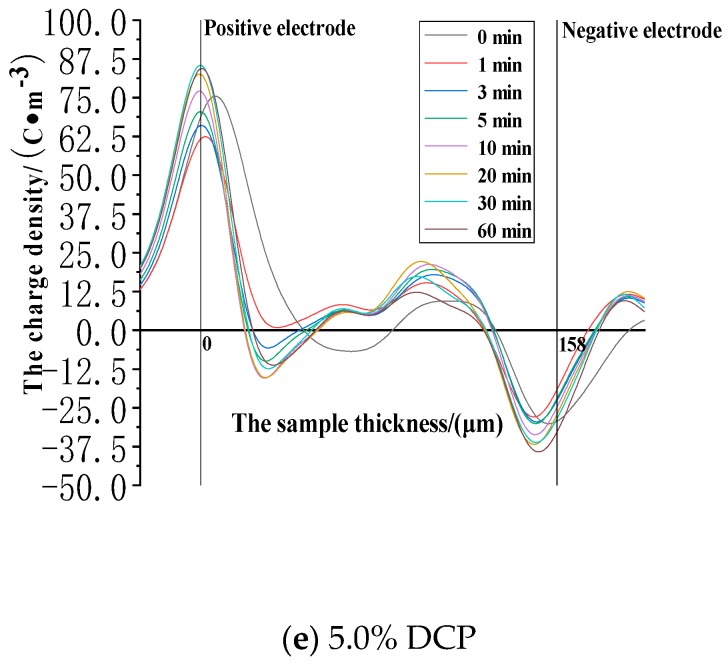
The space charge distribution when voltage is applied to each sample at the electric field strength of 80 kV/mm. (**a**) The experimental results of the pure LDPE samples. (**b**) The experimental results of the samples with 1.0% DCP. (**c**) The experimental results of the samples with 2.1% DCP. (**d**) The experimental results of the samples with 3.0% DCP. (**e**) The experimental results of the samples with 5.0% DCP.

The space charge distribution at the electric field strength of 80 kV/mm in the applied voltage experiment is shown in Figure 6.

It can be seen from Figure 6 that the values of the charge density peaks at the positive electrode position are as follows. The pure LDPE sample is 48.44 C·m^−3^. The sample with 1.0% DCP is 100 C·m^−3^. The sample with 2.1% DCP is 93.82 C·m^−3^. The sample with 3.0% DCP is 74.75 C·m^−3^. And the sample with 5.0% DCP is 85.24 C·m^−3^. The values of the charge density peaks at the negative electrode position are as follows. The pure LDPE sample is 7.62 C·m^−3^. The sample with 1.0% DCP is 34.75 C·m^−3^. The sample with 2.1% DCP is 50.17 C·m^−3^. The sample with 3.0% DCP is 40.08 C·m^−3^. And the sample with 5.0% DCP is 39.18 C·m^−3^. Namely, these values are first increased and then decreased, while the corresponding values at the electric field strength of 30 kV/mm and 50 kV/mm are first reduced and then increased. At the same time, the charge density at the positive electrode position is about 15 C·m^−3^ at the electric field strength of 30 kV/mm, and the charge density at the positive electrode position is 50 C·m^−3^ at the field strength of 50 kV/mm. If the charge density value at the positive electrode position and the electric field strength value increase linearly, the charge density value at the positive electrode position at the field strength of 80 kV/mm should be approximate 40 C·m^−3^. The measured value of the LDPE sample is 48.09 C·m^−3^, but the charge density value of the sample after cross-linking has been much higher than this level. It can be inferred from the above two points that when the electric field strength exceeds 50 kV/mm and reaches 80 kV/mm, the manner of the charge injection and accumulation in the sample after cross-linking should undergo an important change. At the same time, at the electric field strength of 80 kV/mm, only the charge density values of the pure LDPE at the two electrode positions gradually decrease with the experiment of applied voltage continues, and the charge density values in most of the regions of the medium gradually increase with the experiment of applied voltage continues, which is the same as the regulation of the lower electric field strength of 30 kV/mm and 50 kV/mm. In addition, it can be seen from Figure 6 that the charge density values at the electrode positions and inside the medium of the sample after cross-linking show a dramatic fluctuation with the applied voltage experiment continues, and the positions of the charge peaks in the space charge distribution measured at different times also have a large change. This shows that the cross-linking effect does significantly change the charge injection and accumulation characteristics of XLPE. And the reason for the above two phenomena is probably because the small space charge packet phenomenon began to appear in the cross-linking sample at the electric field strength of 80 kV/mm. 

At the electric field strength of 80 kV/mm, the accumulated charges in the pure LDPE sample are still positive, while the cross-linking sample has negative charge accumulation and a significant negative charge density peak. This also confirms the previous judgment that the cross-linking effect introduces the negative charge traps into LDPE. In addition, at the field strength of 80 kV/mm, the charge density of the LDPE sample is much smaller than that of each sample after cross-linking in a large area inside the sample. At the electric field strengths of 30 kV/mm and 50 kV/mm, the charge densities of the pure LDPE are much larger than that of the sample after cross-linking in a large area within the sample.

In summary, the impact of the cross-linking effect on the charge injection and accumulation characteristics of XLPE with different cross-linking degrees is as follows:

(1) The cross-linking effect introduces the negative charge traps into LDPE.
(2) When the electric field strength is low, it is easier for the charges to inject and accumulate in the pure LDPE; this process is difficult at first, and then becomes easy with the increasing content of the cross-linking agent.
(3) When the electric field strength is high, the charges make it easier for XLPE to be injected and accumulated; this process is easy at first and then becomes difficult with the increasing content of the cross-linking agent.

### 3.3. The Impact of the Cross-Linking Effect on the Charge Dissipation Characteristics of XLPE

#### 3.3.1. The Dissipation Characteristics of Space Charge after the Action of 30 kV/mm

**Figure 7:**
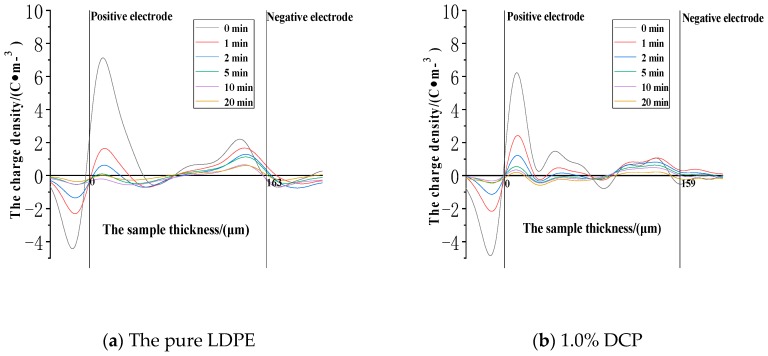

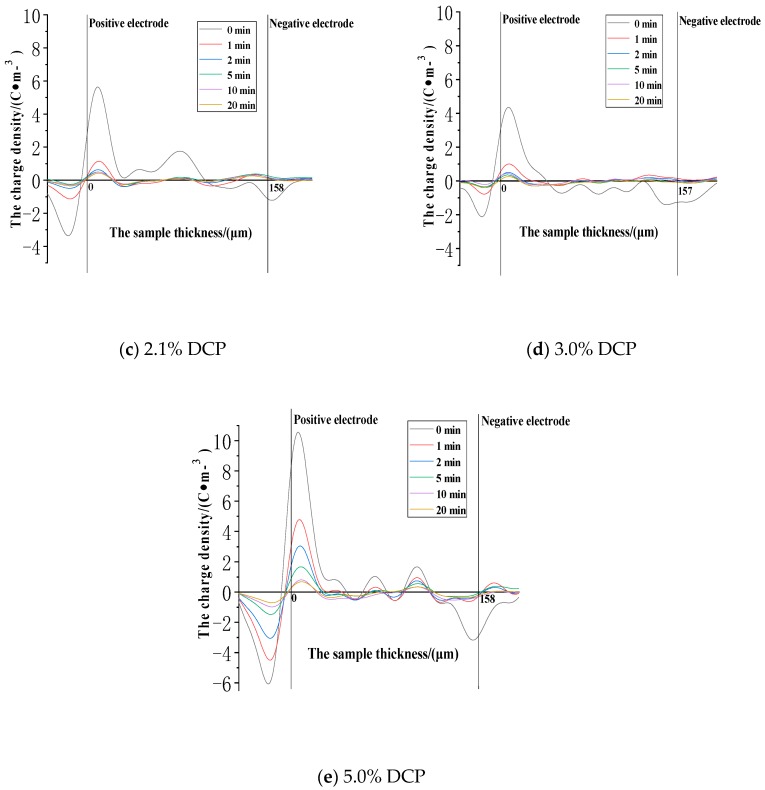
The space charge distribution when each sample experiences de-voltage after the action of 30 kV/mm. (**a**) The experimental results of the pure LDPE samples. (**b**) The experimental results of the samples with 1.0% DCP. (**c**) The experimental results of the samples with 2.1% DCP. (**d**) The experimental results of the samples with 3.0% DCP. (**e**) The experimental results of the samples with 5.0% DCP.

The space charge distribution in the removed voltage experiment after the action of 30 kV/mm is shown in Figure 7.

It can be seen from Figure 7 that some of the charges trapped inside each sample are not immediately removed from the traps and dissipated after the voltage is removed. As the time of the removed voltage is prolonged, the positive and negative space charges are slowly dissipated, which are basically dissipated after approximate 20 min. After the voltage is removed by the short circuit method, the residual charges in the medium can induce the charges of opposite polarity at the electrode positions. The maximum charge density peak in each sample is positive and appears on the side close to the positive electrode, for which there may be two reasons, on the one hand, the position closed to the side of the positive electrode is more favorable for the injection and accumulation of positive charges, which causes a large number of positive charges to be captured by the charge traps at the position closed to the side of the positive electrode, on the other hand, it may be that the position of the negative electrode is relatively far from the sensor, and the acoustic pulse signals inevitably undergo a certain degree of attenuation when propagating in the medium, which also makes the charge density peak near the negative electrode smaller than that near the positive electrode.

It can be also seen from Figure 7 that the positive charge density peaks near the positive electrode of each sample are as follows. The pure LDPE is 7.21 C·m^−3^. The sample with 1.0% DCP is 6.25 C·m^−3^. The sample with 2.1% DCP is 5.68 C·m^−3^. The sample with 3.0% DCP is 4.41 C·m^−3^. And the sample with 5.0% DCP is 10.61 C·m^−3^. It can be seen from the above data that the peak value of the charge density decreases at first and then increases as the content of the cross-linking agent increases. The charge density peak of the sample with 3.0% DCP is the smallest, and the charge density peak is slightly larger than that of the pure LDPE when the content of the cross-linking agent is 5.0%.

It can be seen from Figure 7a that the large positive charge density peaks exist near the positive and the negative electrodes, and these peaks are wider, which penetrate the inside of the medium. Eventually, two charge density peaks occupy a large part of the area in the thickness direction of each sample. In addition, although there is still a positive charge density peak near the positive electrode in Figure 7, the peak is closer to the positive electrode, and the peak is narrower. Additionally, the value of the charge density near the negative electrode is very low, and no significant positive charge peaks appear there.

#### 3.3.2. The Dissipation Characteristics of Space Charge after the Electric Field Strength of 50 kV/mm

**Figure 8:**
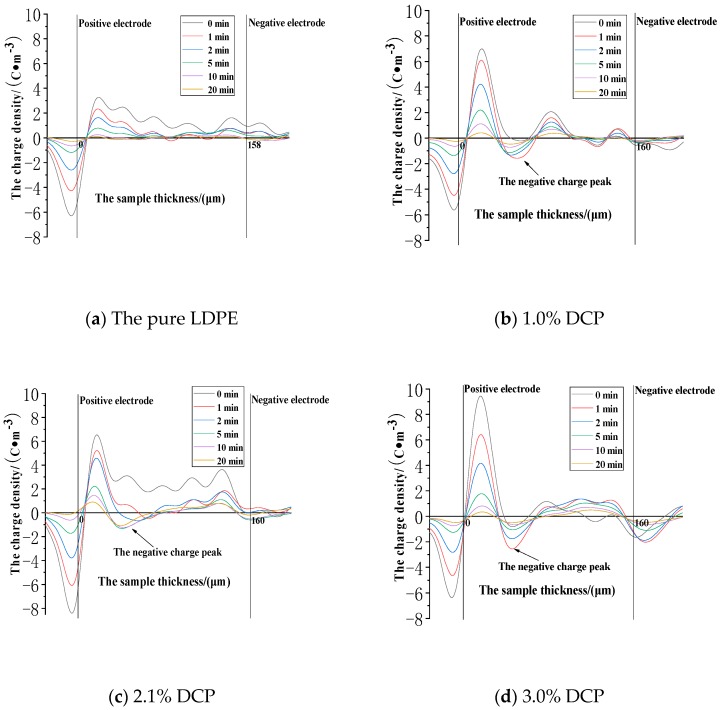

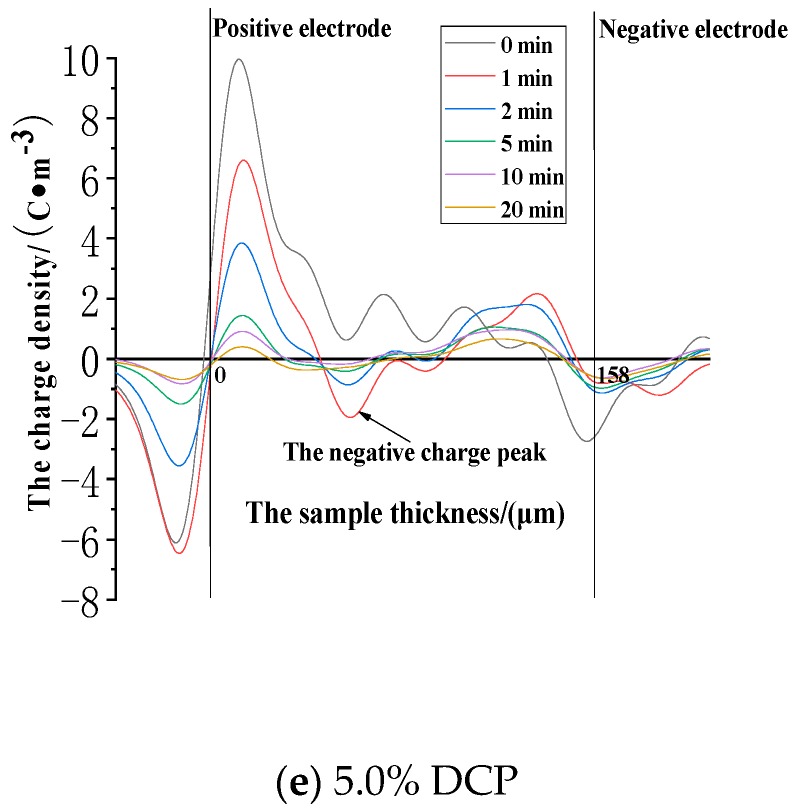
The space charge distribution when each sample experiences de-voltage after the action of 50 kV/mm. (**a**) The experimental results of the pure LDPE samples. (**b**) The experimental results of the samples with 1.0% DCP. (**c**) The experimental results of the samples with 2.1% DCP. (**d**) The experimental results of the samples with 3.0% DCP. (**e**) The experimental results of the samples with 5.0% DCP.

The space charge distribution in the removed voltage experiment after the action of 50 kV/mm is shown in Figure 8.

It can be seen from Figure 8 that the cross-linking effect causes a large change in the distribution characteristics of the residual charges after the voltage is removed. First of all, the charges accumulated in the pure LDPE are all positive, and the maximum peak of the charge densities is below 50 C·m^−3^. A large number of charges are trapped in the entire dielectric region, and the space charge distribution in each sample after cross-linking is not as uniform as the pure LDPE. After cross-linking, the charge distribution in each sample is relatively concentrated near the positive electrode, and the charge density peak of the sample with 5% DCP is even more than 10C·m^−3^. Additionally, the charge distribution of each sample after cross-linking, compared with the charge distribution of the pure LDPE, shows the negative space charge density peak, which indicates that the cross-linking effect introduces the negative charge traps. Eventually, since the charges trapped by the shallow charge traps quickly disengage from the traps and dissipate after the voltage is removed, the charge distribution observed in the short-circuit dissipation experiment is the distribution that the charge are trapped by the deeper charge traps. It can be also seen from Figure 7 that the peak value of the charge density near the positive electrode of the pure LDPE is much smaller than the corresponding value of the sample after cross-linking, which shows that the cross-linking effect greatly increases the densities of the deeper charge traps in LDPE.

Similar to the sample after the action of 30 kV/mm, the internal charges of the sample after the action of 50 kV/mm have a part of the charges trapped by the charge traps as well, and these charges do not immediately disengage from the traps and dissipates. As the removed voltage is prolonged, the positive and negative space charges can slowly dissipate, which can basically dissipate after approximate 20 min. In addition, the maximum positive charge density peak still appears near the positive electrode of each sample. The positive charge density peaks of each sample are as follows. The pure LDPE is 3.34 C·m^−3^. The sample with 1.0% DCP is 6.97 C·m^−3^. The sample with 2.1% DCP is 6.56 C·m^−3^. The sample with 3.0% DCP is 9.49 C·m^−3^. And the sample with 5.0% DCP is 10.02 C·m^−3^. The peak of the charge density shows fluctuation characteristics with the increase of the content of the cross-linking agent, which is different from the corresponding value in the charge distribution decreasing at first and then increasing after the action of 30 kV/mm. Unlike the charge distribution after the action of 30 kV/mm, it is mainly concentrated near the electrode, and there is only a small charge distribution in the deeper part of the medium. The large charge density peak appears in the depth of the inside of the medium, and a large number of charge accumulations are also found in the deep part of the sample after the action of 50 kV/mm.

#### 3.3.3. The Dissipation Characteristics of Space Charge after the Action of 80 kV/mm

**Figure 9:**
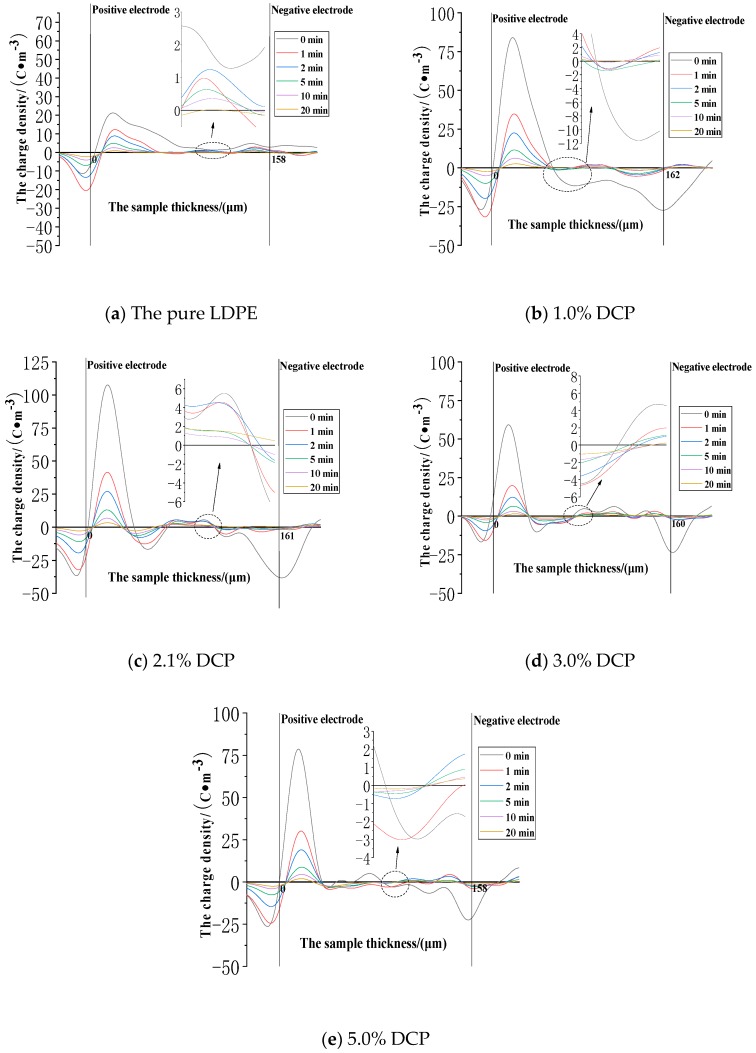
The space charge distribution when each sample experiences de-voltage after the action of 80 kV/mm. (**a**) The experimental results of the pure LDPE sample. (**b**) The experimental results of the samples with 1.0% DCP. (**c**) The experimental results of the samples with 2.1% DCP. (**d**) The experimental results of the samples with 3.0% DCP. (**e**) The experimental results of the samples with 5.0% DCP.

The space charge distribution in the removed voltage experiment after the action of 80 kV/mm is shown in Figure 9.

It can be seen from Figure 9 that some of the internal charges trapped by the deeper charge traps of the medium are not immediately removed from the traps and dissipated after the action of 80 kV/mm. The main positions of the charges trapped by the charge traps in each sample are near the two electrodes, and the maximum positive charge density peaks appearing near the positive electrode are respectively as follows. The pure LDPE is 21.11 C·m^−3^. The sample with 1.0% DCP is 83.93 C·m^−3^. The sample with 2.1% DCP is 7.79 C·m^−3^. The sample with 3.0% DCP is 59.84 C·m^−3^. And the sample with 5.0% DCP is 78.69 C·m^−3^. As the content of the cross-linking agent increases, the charge density increases at first and then decreases, and the maximum value appears in the sample with 2.1% DCP, which is different from the corresponding value in the charge distribution decreasing at first and then increasing after the action of 30 kV/mm. Additionally, the peak value of the charge density near the positive electrode in the removed voltage experiment after the action of 80 kV/mm is much larger than the corresponding value after the action of 30 kV/mm and 50 kV/mm, respectively.

Next, we further analyze the dissipation characteristics of the charges trapped by the charge traps in a large area inside the sample after the action of 80 kV/mm. The areas of the smallest charge density values are magnified in Figure 9, and the features in the areas are observed. It can be found in Figure 9 that although these are the regions with the lowest charge densities in the middle of the sample, the charge density values are still large, which can be compared to the peak charge densities in the entire dielectric region after the action of 30 kV/mm or even 50 kV/mm. That is to say, although the charges trapped by the charge traps after the action of 80 kV/mm are mainly distributed in the regions of the two electrodes, a large number of charges are still distributed in a large area in the deep portion of the sample. Furthermore, the charge densities in these regions can be compared with the maximum charge density in the entire dielectric region after the action of 30kV/mm or even 50kV/mm. At the same time, it can be clearly seen from Figure 9 that the charges trapped by the charge traps in the entire region of the pure LDPE are all positive charges. However, there are negative charges remaining in each sample after cross-linking, and at this time, the negative charge density peaks start to appear. This confirms the conclusion that the cross-linking effect introduces the negative charge traps into the pure LDPE.

In summary, the impact of the cross-linking effect on the dissipation characteristics of the residual charges after the voltage is removed is as follows:

(1) The cross-linking effect increases the density of the deeper charge traps in LDPE.
(2) When the electric field strength of the applied voltage is low, more space charges can remain in the pure LDPE after the voltage is removed. And a number of residual charges decrease at first and then increase with the increase of the content of the cross-linking agent. At the same time, more space charges can remain in XLPE after the voltage is removed. A number of residual charges increase at first and then decrease with the increase of the content of the cross-linking agent when the electric field strength of the applied voltage is high.
(3) The cross-linking effect makes the residual charges more uneven after the voltage is removed, and these charges are mainly distributed in the shallower surface of the medium near the electrode positions.

### 3.4. The Impact of Cross-Linking Effect on Average Charge Density of XLPE

#### 3.4.1. The Charge Injection and Accumulation Characteristics in the Applied Voltage Experiment

**Figure 10:**
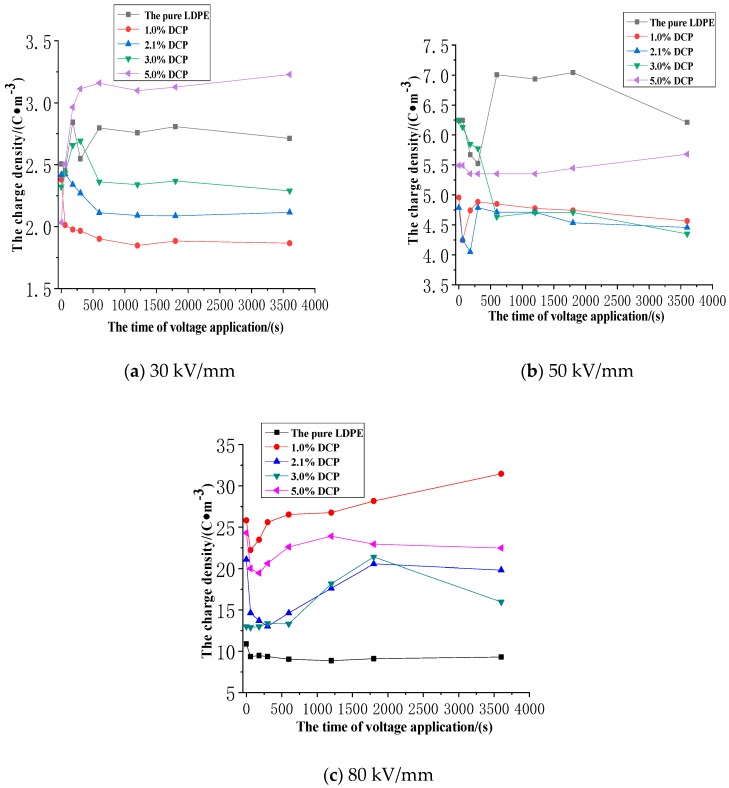
The average charge density of each unaged sample in the applied voltage experiment of different electric field strengths with the time of voltage application. (**a**) The experimental results at the electrical field strength of 30 kV/mm. (**b**) The experimental results at the electrical field strength of 50 kV/mm. (**c**) The experimental results at the electrical field strength of 80 kV/mm.

The average charge density of each unaged sample in the applied voltage experiment of different electric field strengths with the time of voltage application is shown in Figure 10.

It can be seen from Figure 10a–c that the average charge density in each sample gradually increases as the electrical field strength increases, and the average charge density is, respectively, approximate 2.5 C·m^−3^, 5.0 C·m^−3^, and 20 C·m^−3^ at the electrical field strengths of 30kV/mm, 50kV/mm, and 80kV/mm. It can be also seen from Figure 10a–c that the accumulated average charge density is significantly enhanced in the sample when the electrical field exceeds 50 kV/mm.

It can be seen from Figure 10a that when the electrical field strength is 30 kV/mm, the average charge density in each sample is basically similar that of the beginning of voltage application, and then reaches different levels as the time of the voltage application increases. At the beginning of the voltage application, the average charge density tends to stabilize soon after a small fluctuation. During most of the time of the voltage application experiment, the average charge density is at a relatively fixed level with only minor changes. While the average charge density tends to be stable, the average charge density of each sample decreases at first and then increases with the increase of the content of the cross-linking agent, and the average charge density value of the sample is the smallest when the content of the cross-linking agent is 1.0%. However, the average charge density in the sample is the highest when the content of the cross-linking agent is 5.0%, and the average charge density in the sample of the pure LDPE is also at a relatively high level.

It can be seen from Figure 10b that when the electrical field strength is 50 kV/mm, the average charge density in each sample is different at the start of voltage application, which may be caused by the rapid alteration of the charge distribution in the sample when the voltage is applied. However, the initial value of the applied voltage of each sample captured by the measurement system has randomness within a certain time range. At the initial stage of voltage application, the average charge density in each sample fluctuates drastically, whose severity is significantly higher than that when the electrical field strength is low. Moreover, during the whole voltage application experiment, the average charge density in the sample fluctuates greatly, which indicates that it takes longer for the average charge density to reach equilibrium at the electrical field strength of 50kV/mm. During most time of the voltage application experiment, as the content of the cross-linking agent increases, the average charge density in each sample also decreases at first and then increases. The average charge density of the samples with the cross-linking agents of 1.0%, 2.1%, and 3.0%, respectively, is basically similar, and at this time, the average charge density of the pure LDPE is the largest.

It can be seen from Figure 10c that when the electrical field strength is 80 kV/mm, the average charge density in each sample is different at the beginning of voltage application, for the same reason as above. At the beginning of the voltage application experiment, the average charge density in each sample also changes very sharply, and they all have a short period of reducing at first and then increasing. It can be also clearly seen from Figure 10c that during the entire applied voltage experiment, the average charge density in the sample doesn’t tend to be stable, but rather is in a state of volatility or a significant increase. During most of the time of the voltage application experiment, the average charge density in the sample shows the trend of increasing at first and then decreasing as the content of the cross-linking agent increases, which is completely opposite to the variation of the average charge densities at the electrical field strengths of 30 kV/mm and 50 kV/mm. At this same time, the average charge density in the pure LDPE is much lower than that in the sample after cross-linking.

In summary, we can infer that when the electrical field strength exceeds a certain value, the mechanism of the space charge injection and accumulation in XLPE undergoes some changes from quantitative changes to qualitative changes. This article has given an explanation for this macroscopic phenomenon from the microscopic point of the sample in Section 3.5.

#### 3.4.2. The Charge Dissipation Characteristics in the Removed Voltage Experiment

**Figure 11:**
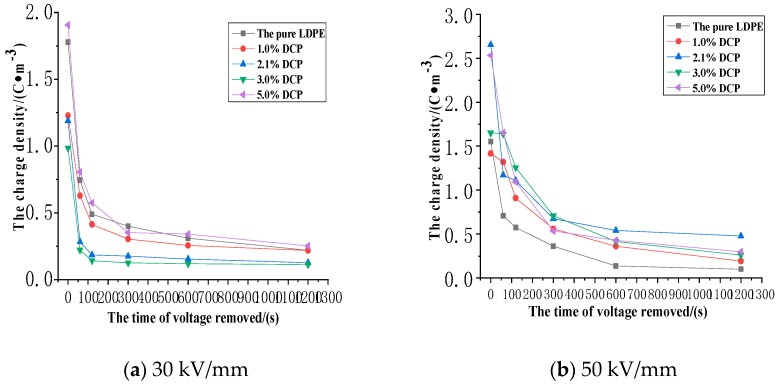

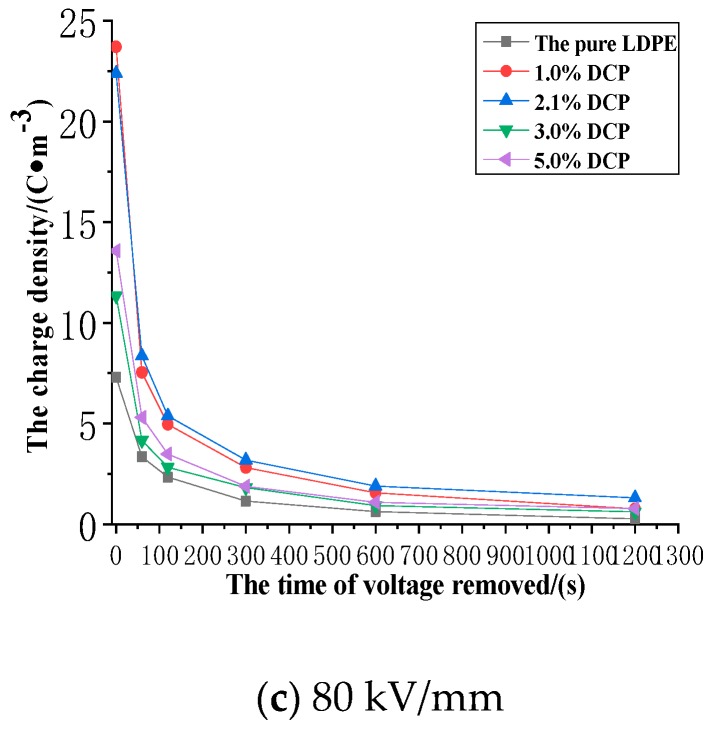
The average charge density of each unaged sample in the removed voltage experiment of different electric field strengths with the time of voltage application. (**a**) The experimental results at the electrical field strength of 30 kV/mm. (**b**) The experimental results at the electrical field strength of 50 kV/mm. (**c**) The experimental results at the electrical field strength of 80 kV/mm.

The average charge density of each unaged sample in the removed voltage experiment of different electric field strengths in relation to the time of voltage application is shown in Figure 11.

It can be seen from Figure 11a–c that as the removed voltage increases, the charges remaining in the sample gradually dissipate. At the beginning of the removed voltage, the residual charges dissipate faster, and the residual charge dissipation rate is slower in the late stage of the removed voltage. Based on the above analysis, it can be seen that the dissipation of residual charges is basically in accordance with the discipline of exponential decay.

It can be also clearly seen that as the applied electrical field strength in the applied voltage experiment increases, the average density of the charges trapped by the charge traps in each sample gradually increases in the erase voltage experiment. The average charge density after the action of 30 kV/mm, 50 kV/mm, and 80 kV/mm, respectively, is approximate 1.5 C·m^−3^, 2 C·m^−3^, and 15 C·m^−3^. It can be found that when the value of the electrical field strength exceeds 50 kV/mm, the average density of residual charges in the sample also increases significantly, which also strongly supports the previous inferences. Namely, the mechanism of the space charge injection and accumulation in XLPE undergoes some changes from quantitative changes to qualitative changes when the electrical field strength exceeds a certain value.

It can be seen from Figure 11a that the average charge density of each sample decreases at first and then increases with the increase of the content of the cross-linking agent in the removed voltage experiment after the action of 30 kV/mm. In addition, the average charge density of the sample with 3% DCP is the smallest, while the average charge densities of the pure LDPE and the sample with 5% DCP are at relatively high levels.

It can be seen from Figure 11b that the average charge density of each sample fluctuates as the content of cross-linking agent increases after the action of 50 kV/mm. However, its overall trend is to increase at first and then decrease, which is different from the alter discipline at 30 kV/mm, and it can be clearly seen that the charge density of the pure LDPE is the smallest.

It can be seen from Figure 11c that a large number of space charges are retained in each sample in the removed voltage experiment after the action of 80 kV/mm. With the increase of the content of the cross-linking agent, the average charge density of each sample shows an increase at first and then a decrease as well. The average charge density in the sample is the largest when the content of the cross-linking agent is 2.1%, and the charge density in the pure LDPE is the smallest.

#### 3.4.3. The Decay Model of the Average Charges

It can be seen from Figure 11 that there may be a certain relationship between the residual charges of each sample and the time in the removed voltage experiment. In order to obtain the decay discipline of residual charges of each sample with time, at the same time, in order to obtain the dissipation characteristics of space charges with time as well, we built the relational model as shown in Equation (2) based on a large number of experiments [6,7,8,9,10,11,12,13,14,15].

Namely, the model can be expressed as:

(2) $$Q(t)=Q_{0}+\alpha e^{-\frac{t}{\beta }}$$

In this formula, Q(t) represents the average charge density, which is a function of time *t*. $Q_{0}$ represents the number of residual space charges when the attenuation of the average charge density tends to be stable. α represents the attenuation of the average charge density, namely, the difference between the initial value and the stable value. β represents the decay time constant of the average charge density.

In order to verify the correctness of the theoretical model, the theoretical results obtained by the above model were compared with the real experimental results.

**Figure 12:**
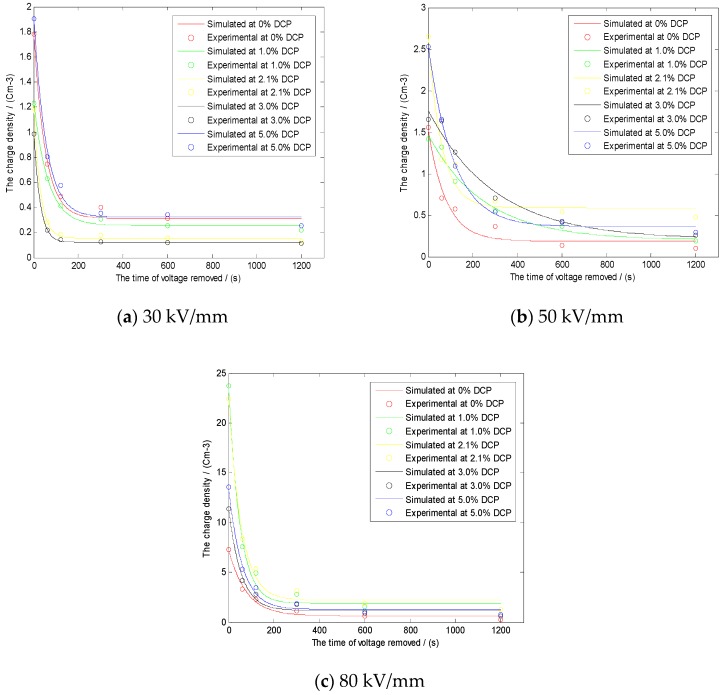
The comparison between the theoretical results obtained by the above model and the real experimental results after the action of, respectively, 30 kV/mm, 50 kV/mm, and 80 kV/mm. (**a**) The comparison after the action of 30 kV/mm. (**b**) The comparison after the action of 50 kV/mm. (**c**) The comparison after the action of 80 kV/mm. (the sample with 0%DCP in the legend is the pure LDPE).

After the action of 30 kV/mm, 50 kV/mm, and 80 kV/mm, respectively, the comparison between the theoretical results obtained by the above model and the real experimental results is shown in Figure 12.

The values of R^2^ in Figure 12a–c can be obtained by comparing the theoretical simulation results with the real experimental data by MATLAB software, whose specific values are as follows.

In Figure 12a, the values of R^2^ are, respectively, 0.9894, 0.9955, 0.9980, 0.9999, and 0.9950 from the sample with 0% DCP to the sample with 5% DCP. In Figure 12b, the values of R^2^ are, respectively, 0.9894, 0.9785, 0.9692, 0.9799, and 0.9989 from the sample with 0% DCP to the sample with 5% DCP. In Figure 12c, the values of R^2^ are, respectively, 0.9883, 0.9989, 0.9958, 0.9900, and 0.9925 from the sample with 0% DCP to the sample with 5% DCP. 

It can be seen from the above values of R^2^ that the variation discipline of R^2^ is in good agreement with the alteration of the corresponding physical characteristics in the actual charge distribution, which indicates that the model is effective for explaining the dissipation of space charges in the removed voltage experiment. Then, in the next experiments, the space charge data of the samples are processed by the model, and the obtained parameters are analyzed; finally, the corresponding information can be obtained. We can also conduct preliminary theoretical analysis through this theoretical model. For example, when the value of α is large, it means that more charges remain in the sample after the voltage is removed. Additionally, when the value of β is large, it means that the charge dissipation rate in the sample is slow after the voltage is removed. At the same time, it can be also inferred that the accumulation positions of the charges are deeper or the charge traps in the sample are deeper.

### 3.5. The Microscopic Interpretation of the Cross-Linking Effect on Space Charge Characteristics

In the above, the variation of the average space charge densities in the applied voltage experiment and the removed voltage experiment of each unaged sample under different electric field strengths are analyzed, and the following conclusions are obtained:

(1) In the applied voltage experiment, the average charge densities of the samples decrease at first and then increase with the increase of the content of the cross-linking agent at the electric field strengths of 30 kV/mm and 50 kV/mm, while the average charge density increases at first and then decreases at the electric field strength of 80 kV/mm.
(2) In the removed voltage experiment, the average charge density in each sample decreases at first and then increases with the increase of the content of the cross-linking agent at the electric field strength of 30 kV/mm, and the average charge densities increase at first and then decrease at the electric field strengths of 50 kV/mm and 80 kV/mm.
(3) Compared to the lower electric field strength, the average charge density in each sample increases significantly at the electric field strength of 80 kV/mm.
(4) The threshold field strength of each unaged sample is less than 30 kV/mm, and this shows the trend of increasing at first and then decreasing as the content of the cross-linking agent increases.

**Figure 13:**
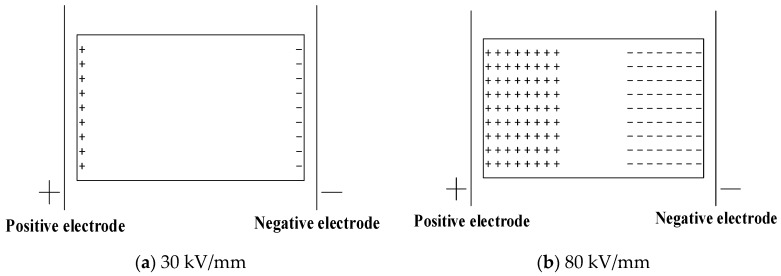
The situations of the charge injection and migration. (**a**) The schematic of charge injection and migration (30 kV/mm). (**b**) The schematic of charge injection and migration (80 kV/mm).

The reason for these macroscopic phenomena may be that the cross-linking effect causes more chain breaks and end groups in LDPE; thereby, this increases the number of deeper charge traps in LDPE. The charge injection and migration process is shown in Figure 13.

It can be seen from Figure 13a that the charge traps in the medium near the electrodes capture a certain number of charges after the DC electrical field is applied, and if the applied electrical field strength is low at this time, the charges cannot easily escape from the traps and migrate to the inside of the medium (it can be seen clearly in Figure 6 that the charges of each cross-linked sample are mainly distributed near the surface of the medium after the voltage is removed, while a large number of charges still exist in the depth of the pure LDPE sample). Additionally, the charges trapped by the traps have a certain shielding effect on the electrodes, reducing the electrical field strength at the surfaces of the electrodes and the medium, so that the charges are less likely to be injected. Therefore, the result leads to an increase of the threshold electrical field strength of the medium, and the number of charges in the medium is relatively small when the voltage is applied and after the voltage is removed.

It can be seen from Figure 13b that the charge traps in the medium near the electrodes still capture a certain number of charges, and this also has a shielding effect on the electrodes when the electrical field strength is very high. However, because the applied electrical field strength is very high, the results in the actual electrical field strength values in the local range of the sample surface are larger than the threshold field strength values of the sample, even if there is a shielding effect of the charges trapped by the traps near the surface of the sample, and there will still be a amount of charge injection and accumulation inside the medium. On the other hand, because the applied electrical field strength is very high, this causes the trapped charges to gradually get out of the traps and migrate deeper into the interior of the medium, resulting in a large number of trapped charges in the medium away from the electrode positions (it can be seen clearly in Figure 8 that a large number of charges exist in the range of the entire thickness of the medium after the voltage is removed. Even at this time, the lowest charge density in the sample is larger than the maximum charge density in the charge distribution at 30 kV/mm). This causes the average charge density value in the cross-linked sample to be greater than the charge density value in the pure LDPE in the applied voltage experiment and the dissipative experiment in which the applied electrical field strength is very high. Besides, the average charge density value of the sample can increase significantly because there are a large number of charges trapped by the traps in a large area inside the sample.

When the applied electrical field strength is at a certain value in the middle of the above two voltage values—for example, 50 kV/mm, compared with the charge density in the pure LDPE sample—the average charge density in the cross-linked sample is relatively small when the voltage is applied. Furthermore, the average charge density in the sample after cross-linking is relatively large when the voltage is removed. This may be because when the applied electrical field strength is at a relatively high value, the charges trapped by the charge traps near the surface of the sample can be out of the traps once they are slightly disturbed and gradually move deeper into the sample. This also weakens the impact of the shielding effect, so that more charges can be injected into the sample. There are many charges trapped by the charge traps in the relatively deep part of the sample, which results in a larger average charge density in the cross-linked sample in the removed voltage experiment. However, when the voltage is applied, since the pure LDPE sample has a large number of charges trapped by the shallow traps in the entire medium (the charges are dissipated from the traps in a short time after the voltage is removed by the short circuit method), therefore, the average charge density in the pure LDPE sample is still relatively large in the applied voltage experiment.

As the content of the cross-linking agent increases, the threshold field strength of each sample increases at first and then decreases. This may be because [21] when the content of the cross-linking agent is at a low level, as the content of the cross-linking agent increases, the number of free radicals generated by the decomposition of DCP in the reaction system is small. This can effectively induce the formation of long-chain radicals in the LDPE molecular chains. Then, cross-linking terminates between the molecular chains, and the XLPE sample obtained after cross-linking increases the degree of cross-linking. However, when the content of the cross-linking agent is at a high level, the concentration of free radicals in the reaction system exceeds a certain value. Thereby, the probability of collision between free radicals increases, resulting in a decrease in the efficiency of cross-linking. This leads to a decrease in the degree of cross-linking of the XLPE sample obtained after cross-linking. As the content of cross-linking agent increases, the cross-linking degree of XLPE can increase at first and then decrease, which can cause the threshold field strength of each sample to increase at first and then decrease as well.

## 4. Conclusions

In conclusion, the cross-linking effect significantly increases the threshold field strength of XLPE. As the content of cross-linking agent increases, the threshold field strength increases at first and then decreases, and the threshold electrical field strength reaches the maximum value when the content of the cross-linking agent is 1.0% or 2.1%. Additionally, the cross-linking effect introduces the negative charge traps into the LDPE and increases the density of the deeper charge traps. Besides, when the applied electrical field strength is low, the cross-linking effect makes the charges difficult to be injected and accumulated. When the applied electrical field strength is high, the cross-linking effect makes the charges easier to be injected and accumulated. When the voltage is removed, the charges are mainly distributed near the electrodes when the electrical field strength is low. Meanwhile, the charge distribution is deep when the electrical field strength is high. In addition, when the electrical field strength of the applied voltage experiment is low, more space charges can remain in the pure LDPE after the voltage is removed, and the number of residual charges decrease at first and then increase with the increase of the content of the cross-linking agent. When the electrical field strength of the applied voltage experiment is high, more charges can remain in the XLPE with different cross-linking degrees after the voltage is removed, and the number of residual charges increase at first and then decrease with the increase of the content of the cross-linking agent. In addition, we have extracted and analyzed the average charge density as well, which can quantitatively obtain the impact of the cross-linking effect. Namely, when the voltage is applied, the average charge densities at 30 kV/mm and 50 kV/mm decrease at first and then increase with the increase of the content of the cross-linking agent, while the average charge density at 80 kV/mm increases at first and then decreases. After the voltage is removed, the average charge density at 30 kV/mm decreases and then increases with the increase of the content of the cross-linking agent, and the average charge density increases at first and then decreases at, respectively, 50 kV/mm and 80 kV/mm. Additionally, we have also summarized the theoretical model of the average charge decay, namely, $Q(t)=Q_{0}+\alpha e^{-\frac{t}{\beta }}$. We have also compared the theoretical simulation results with the actual experimental results, and we have found that the variation of the theoretical model is in good agreement with the change of the corresponding physical characteristics in the actual charge distribution. This finding indicates that the model is very effective for explaining the dissipation characteristics of the space charges after the voltage is removed.
